# Patients with positive malaria tests not given artemisinin-based combination therapies: a research synthesis describing under-prescription of antimalarial medicines in Africa

**DOI:** 10.1186/s12916-019-1483-6

**Published:** 2020-01-30

**Authors:** Shennae O’Boyle, Katia J. Bruxvoort, Evelyn K. Ansah, Helen E. D. Burchett, Clare I. R. Chandler, Siân E. Clarke, Catherine Goodman, Wilfred Mbacham, Anthony K. Mbonye, Obinna E. Onwujekwe, Sarah G. Staedke, Virginia L. Wiseman, Christopher J. M. Whitty, Heidi Hopkins

**Affiliations:** 1 London School of Hygiene and Tropical Medicine, London, UK; 2 Department of Research and Evaluation, Kaiser Permanente Southern California, Pasadena, USA; 3 Centre for Malaria Research, University of Health and Allied Sciences, Accra, Ghana; 4 Public Health Biotechnology, University of Yaoundé I, Yaoundé, Cameroon; 5 Makerere University School of Public Health, Kampala, Uganda; 6 Department of Pharmacology and Therapeutics, University of Nigeria, Enugu, Nigeria; 7 School of Public Health and Community Medicine, University of New South Wales, Sydney, Australia

**Keywords:** Malaria, Diagnosis, Case management, Fever case management, Rapid diagnostic test, Prescribing, Prescription, Antimalarial, ACT, Antibiotic

## Abstract

**Background:**

There has been a successful push towards parasitological diagnosis of malaria in Africa, mainly with rapid diagnostic tests (mRDTs), which has reduced over-prescribing of artemisinin-based combination therapies (ACT) to malaria test-negative patients. The effect on prescribing for test-positive patients has received much less attention. Malaria infection in endemic Africa is often most dangerous for young children and those in low-transmission settings. This study examined non-prescription of antimalarials for patients with malaria infection demonstrated by positive mRDT results, and in particular these groups who are most vulnerable to poor outcomes if antimalarials are not given.

**Methods:**

Analysis of data from 562,762 patients in 8 studies co-designed as part of the ACT Consortium, conducted 2007–2013 in children and adults, in Cameroon, Ghana, Nigeria, Tanzania, and Uganda, in a variety of public and private health care sector settings, and across a range of malaria endemic zones.

**Results:**

Of 106,039 patients with positive mRDT results (median age 6 years), 7426 (7.0%) were not prescribed an ACT antimalarial. The proportion of mRDT-positive patients not prescribed ACT ranged across sites from 1.3 to 37.1%. For patients under age 5 years, 3473/44,539 (7.8%) were not prescribed an ACT, compared with 3833/60,043 (6.4%) of those aged ≥ 5 years. The proportion of < 5-year-olds not prescribed ACT ranged up to 41.8% across sites. The odds of not being prescribed an ACT were 2–32 times higher for patients in settings with lower-transmission intensity (using test positivity as a proxy) compared to areas of higher transmission. mRDT-positive children in low-transmission settings were especially likely not to be prescribed ACT, with proportions untreated up to 70%. Of the 7426 mRDT-positive patients not prescribed an ACT, 4121 (55.5%) were prescribed other, non-recommended non-ACT antimalarial medications, and the remainder (44.5%) were prescribed no antimalarial.

**Conclusions:**

In eight studies of mRDT implementation in five African countries, substantial proportions of patients testing mRDT-positive were not prescribed an ACT antimalarial, and many were not prescribed an antimalarial at all. Patients most vulnerable to serious outcomes, children < 5 years and those in low-transmission settings, were most likely to not be prescribed antimalarials, and young children in low-transmission settings were least likely to be treated for malaria. This major public health risk must be addressed in training and practice.

**Trial registration:**

Reported in individual primary studies.

## Background

Malaria remains among the top ten causes of death in many African countries, and acute febrile illness is the most common presentation in most African outpatient clinics [1]. Prompt antimalarial treatment with the recommended artemisinin-based combination therapies (ACTs) [2] is highly effective, and especially important in cases of *Plasmodium falciparum* malaria which is responsible for the majority of malaria-related deaths both globally and in Africa [3].

Whilst the probability of aquiring malaria increases with transmission, in many endemic African settings, two groups have been shown to be at particularly high risk of mortality if they acquire malaria: young children (< 5 years) and children of any age in lower-transmission settings where immunity to severe disease is acquired slowly if at all [4]. Cerebral malaria in particular is more common in low-transmission settings [5] often leading to higher mortality rates. In both groups, mortality can be prevented by effective antimalarials if diagnosed early (i.e. in outpatient settings) and mortality in these children should be close to zero if treated promptly when symptoms first start. In contrast to semi-immune adults in high-transmission settings, it is very unlikely that malaria parasitaemia is not relevant to the cause of the fever in young children or those in low-transmission settings, making delayed or absent antimalarial treatment potentially fatal.

In 2010, the World Health Organization (WHO) updated its guidelines to recommend universal parasitological confirmation of malaria before treatment [2]. This policy shift aimed to encourage more rational use of antimalarials by limiting over-diagnosis of malaria and over-prescription of the newer, more expensive ACTs; promote diagnosis of other febrile illnesses in patients without malaria; and slow development of resistance to artemisinin and its partner drugs [2]. The introduction of malaria rapid diagnostic tests (mRDTs) has been key in increasing access to diagnostic testing, particularly in settings where traditional microscopy methods are not widely available [6]. Growing evidence shows that parasitological diagnosis of malaria can be achieved at the community level [7, 8] and in private health care settings [9–13]. Thanks in large part to the introduction of mRDTs, diagnostic testing of suspected malaria cases presenting to public health care facilities in the African region has increased from 33% in 2010–2012 to 59% overall, and higher in many sites, by 2015–2017 [14].

A number of mRDT evaluations have assessed impact on case management, and in most settings, attention has been focused on the potential for mRDT use to improve targeting of antimalarials primarily by reducing over-prescription—i.e. health workers adhere to *negative* test results and do not prescribe antimalarials [7, 15, 16]. The impact on malaria test-*positive* patients, when reported, has generated less concern; for example, a 2017 systematic review showed that in most studies that investigated the appropriateness of treatment following a positive mRDT result, more than 95% of patients received appropriate antimalarial medication, although three studies showed that more than 10% of mRDT-positive patients did not [17]. Whilst reducing unnecessary and inappropriate antimalarial use and identifying non-malarial causes of fever is a major public health goal, it remains essential that those who do have clinical malaria are given timely and effective treatment.

The ACT Consortium, a global research partnership designed to address core themes around malaria case management, conducted studies between 2007 and 2013 to assess the impact of mRDT implementation across varied epidemiological and health care contexts. In these studies, the proportion of patients who had a positive mRDT but were not prescribed an ACT varied widely [18]. Relatively little is known about what might contribute to under-prescription for test-positive patients. This paper presents an analysis of ACT Consortium data, focusing specifically on this potentially dangerous phenomenon which was previously identified across multiple settings [18, 19]. We aim to help define the potential scale of under-prescription and to identify factors associated with ACT non-prescription. Specifically, this analysis examines non-prescription of ACT antimalarials for mRDT-positive patients in two groups that have not been examined but who are more vulnerable to poor outcomes: young children and individuals in lower-transmission settings in whom significant immunity is unlikely and mortality from untreated malaria is often higher.

## Methods

### Studies included in the analysis

ACT Consortium studies were designed collaboratively to form a multifaceted investigation of the impact of interventions to improve the use of ACT antimalarials. This analysis examines in more detail data on patients in ACT Consortium study areas in Africa who tested positive for malaria by mRDT but who were not prescribed ACT antimalarials [18, 19]. The primary studies were designed to measure the impact of mRDT implementation on fever case management across a variety of settings. Studies were included in this analysis if they collected data on patient consultations for suspected malaria, evaluated an intervention to implement mRDTs for use by health care providers, and were conducted in sub-Saharan Africa where the predominant malaria species is *P. falciparum* and the recommended first-line therapy for malaria is an ACT. The eight studies meeting these criteria are described in Tables 1 and 2, including the abbreviation for each study used throughout the text.

**Table 1:** Description of study contexts

Study abbreviation and country		Region (location)	Study dates	Endemicity setting (mRDT positivity)		Number (%) of patients with a positive mRDT result that are under 5 years^†^	Health care sector
				Number (%) of patients with a positive mRDT result of those tested with mRDT	Category		
Cam1 [﻿2﻿0]	Cameroon	West Cameroon (Bamenda)	October–December 2011	124/598 (20.7)	Mod–low	34/124 (27.4)	Public/mission
		Central Cameroon (Yaoundé)		145/390 (37.2)	Mod–low	62/145 (42.8)	
Ghan1 [﻿21﻿]	Ghana	Southeast Ghana (Dangme West)	August 2007–December 2008	1308/3631 (36.0)	Mod–low	407/1308 (31.1)	Public
Nige1 [﻿24﻿]	Nigeria	South central Nigeria (Udi)	July–December 2009; June–December 2011	139/323 (43.0)	Mod–high	16/137 (11.7)	Public and private retail
		South central Nigeria (Enugu)		442/788 (56.1)	High	28/434 (6.5)	
Tanz1 [﻿23﻿]	Tanzania	West Tanzania (Mbeya)	May–October 2010; April–July 2012	18/128 (14.1)	Low	9/18 (50.0)	Public
		North Tanzania (Mwanza)		46/278 (16.6)	Low	34/46 (73.9)	
		Southeast Tanzania (Mtwara)		173/367 (47.1)	Mod–high	110/173 (63.6)	
Tanz2 [﻿22﻿]	Tanzania	Northeast Tanzania (Kilimanjaro)	September 2010–January 2011	295/4334 (6.8)	Low	51/294 (17.3)	Public
		Northeast Tanzania (Tanga)	February 2011–March 2012	4105/12,963 (31.7)	Mod–low	1429/4102 (34.8)	
Uga1 [﻿25﻿]	Uganda	Southeast Uganda (Tororo)	April 2011–March 2013	90,269/132,241 (68.3)	High	37,339/88,875 (42.0)	Public
Uga2 [﻿26﻿]	Uganda	Southwest Uganda (Nyakishenyi)	January–December 2011	37/1128 (3.3)	Low	4/35 (11.4)^*^	Community health worker
		Southwest Uganda (Bwambara)		3411/7632 (44.7)	Mod–high	238/3342 (7.1)^*^	
Uga3 [﻿27﻿]	Uganda	South central Uganda (Mukono)	January–December 2011	5690/9987 (57.0)	High	2239/5597 (40.0)	Private retail

*Uga2 included only patients aged < 6 years; proportions presented for patients aged < 1 year

^†^Denominators vary to reported number testing positive by mRDT due to missing data for age. Nige1 (Udi: *n* = 2; Enugu: *n* = 8), Tanz2 (Kilimanjaro: *n* = 1; Tanga: *n* = 3), Uga1 (*n* = 1394), Uga2 (Bwambara: *n* = 22), Uga3 (*n* = 28)

**Table 2:** Description of study design and interventions

Project site	Study design	Primary study intervention arms	Categorisation of intervention arms used in this study
Cam1	Cluster randomised trial	Basic intervention: 1-day training on malaria diagnosis, mRDTs, and prescribing antimalarials. Enhanced intervention: additional 2-day training on adapting to guideline changes, identifying alternative causes of febrile illnesses, and communication skills. (Primary study included control group with no training or receipt of mRDTs)	No/basic training: includes those from the basic intervention arm
			BC arm: includes those from the enhanced training arm
Ghan1	Individually randomised trial	Intervention: 2-day training in sensitivity and specificity of mRDTs, performing mRDTs, prescribing antimalarials, identifying alternative causes of febrile illnesses, and refresher on national guidelines. (Primary study included control group practising current standard of care: presumptive diagnosis (clinical setting) or microscopy (microscopy setting))	No/basic training: includes those from the intervention arm (NB comparison group did not use mRDTs, and was therefore excluded from this analysis)
Nige1	Cluster randomised trial	Control: half-day demonstration on use of mRDTs, plus receipt of pictorial aid. Basic intervention: 2-day training on performing mRDTs, prescribing antimalarials, and communication skills. Enhanced intervention: additional community sensitisation element including teacher/student education for malaria awareness. (Primary study also included a formative study)	No/basic training: includes those from the control arm
			BC arm: includes those from the basic intervention arm
			BC + CS arm: includes those from the enhanced intervention arm
Tanz1	Observational study (during national rollout of mRDTs)	Intervention: 2-day government training on performing mRDTs, prescribing antimalarials, rationale for guideline change, and identifying alternative causes of febrile illnesses. (Primary study included baseline and endline surveys for evaluation)	No/basic training: includes those that undertook government training (NB comparison group did not use mRDTs, and was therefore excluded from this analysis)
Tanz2	Baseline survey followed by cluster randomised trial	Control: 2-day government training on performing mRDTs, prescribing antimalarials, rationale for guideline change, and identifying alternative causes of febrile illnesses. Intervention: three half-day workshops on adapting to and sustaining guideline changes, and communication skills. Enhanced intervention: as above (intervention) plus receipt of additional visual communication resources for patients and facilities. (Primary study included a pilot study with 1-day basic training on mRDT use)	No/basic training: includes those from control arm
			BC arm: includes those from the intervention arm
			BC + CS arm: includes those from the enhanced intervention arm
Uga1	Cluster randomised trial	Intervention: 2-day training on performing mRDTs, prescribing antimalarials, identifying alternative causes of febrile illnesses, and communication skills. (Primary study included patients in the control group tested by mRDT if already available in health care facility, but training on use and interpretation of mRDTs not supplied by study)	No/basic training: includes those from the control arm that were tested by mRDTs not supplied by the study
			BC arm: includes those from the intervention arm
Uga2	Cluster randomised trial	Intervention: 4-day training on performing and reading mRDTs, prescribing antimalarials, dealing with negative cases, communication skills, community sensitisation for diagnostic testing, plus visual communication resources for health care workers. (Primary study included control group receiving 3-day training in malaria diagnosis and referral (but not in use of mRDTs), and community sensitisation for diagnostic testing)	BC + CS arm: includes those from the intervention arm (NB comparison group did not use mRDTs, and was therefore excluded from this analysis)
Uga3	Cluster randomised trial	Intervention: 4-day training for drug shop vendors in performing and reading mRDTs, prescribing antimalarials, dealing with negative cases, communication skills, and community sensitisation for diagnostic testing. (Primary study included formative study, and control group receiving 3-day training in malaria diagnosis and referral (but not in use of mRDTs), and community sensitisation for diagnostic testing)	BC + CS arm: includes those from the intervention arm (NB comparison group did not use mRDTs, and was therefore excluded from this analysis)

*BC* enhanced training arm with behaviour change component, *BC + CS* enhanced training arm with behaviour change and community sensitisation components

The eight studies included in this analysis were conducted between 2007 and 2013 in Cameroon, Ghana, Nigeria, Tanzania, and Uganda [20–27]. The studies assessed the introduction of mRDTs among health care providers in public health centres (Cam1, Ghan1, Tanz1, Tanz2, Uga1), public and private facilities (Nige1), private drug shops (Uga3), and community health programmes (Uga2). Seven studies were designed as cluster randomised trials, and one as an observational study carried out before and after a national rollout of mRDTs in government-sponsored primary care facilities (Tanz1). Most studies recorded data on all outpatients presenting with suspected malaria, one study included only children aged under 6 years (Uga2), and two studies collected data on all outpatient consultations (Tanz2, Uga1). For the purposes of this analysis, patients not tested by mRDT, and those with a negative mRDT result, were excluded. Data were collected through provider-completed registers (Ghan1, Uga1, Uga2, Uga3), patient exit interviews (Tanz1), or a combination of both methods (Cam1, Nige1, Tanz2).

### Outcome description

The analysis investigated the following: (i) proportion of patients with a positive mRDT result who were not prescribed ACT, (ii) patient and provider characteristics associated with non-prescription of ACT for mRDT-positive patients, and (iii) other medications prescribed for patients with positive mRDT results who did not receive ACT.

Primary studies varied in design, context, and implementation, and therefore, not all variables considered for this analysis were available for every study. Patient age and sex, mRDT test result, and antimalarial prescription (ACT and non-ACT) were recorded in all studies. Because of the potential severity of malaria in children under 5 years [28], patients were grouped into binary age categories of under 5 years (< 5) or 5 years and over (≥ 5) to explore the effect of age within each study setting. The exception was Uga2: as Uga2 only included patients under 6 years, the binary age groups for this study were categorised as under 1 year (< 1) and 1 year and over (≥ 1). Among children under age 5 years, newborns and infants aged less than 12 months are most vulnerable to malaria, with increased risk of rapid disease progression, severe anaemia, and death [29].

Three studies were conducted in a single geographic area (Ghan1, Uga1, Uga3), and five studies were conducted in multiple locations (Cam1, Nige1, Tanz1, Tanz2, Uga2) with differing malaria endemicity. To allow comparison of prescribing practices within primary studies that were conducted in more than one endemic zone, the proportion of patients testing mRDT-positive (out of all patients tested) at each site was used as a proxy for malaria endemicity. Study settings were grouped into four categories: low positivity, 0 to 19.9% patient mRDT results positive (Tanz1, Tanz2, Uga2); moderate-to-low positivity, 20.0 to 39.9% (Cam1, Ghan1, Tanz2); moderate-to-high positivity, 40.0 to 54.9% (Nige1, Tanz1, Uga2); and high positivity, ≥ 55.0% (Nige1, Uga1, Uga3). For simplicity, this proxy estimate is referred to as “endemicity” in the remaining text.

Table 2 describes mRDT training and intervention design in each study site. In four primary studies, prescribers in control arms did not use mRDTs and continued with their current method of diagnosis (e.g. microscopy, or clinical judgement; Ghan1, Uga1, Uga2, Uga3). These arms were excluded from this analysis. However, a number of patients in the control arm of one study (Uga1: *n* = 8910) were tested using mRDTs not supplied through the primary studies; analysis with and without this subgroup found no difference in overall results.

All primary studies included at least one intervention arm that introduced mRDTs, and some studies included an additional intervention arm where mRDT introduction was coupled with enhanced health worker training and elements of community involvement. For the analyses presented here, study interventions were re-categorised to allow comparison of prescription practices across the training groups (Table 2): (i) no or basic training—mRDTs introduced with either no training or basic health worker training on how to perform the mRDT and interpret the result; (ii) enhanced training with behaviour change (BC arm)—mRDTs introduced with training on mRDT use and interpretation of results; and (iii) enhanced training with behaviour change plus community sensitization (BC + CS arm)—BC training as above plus an element of community involvement. Uga2 and Uga3 included availability of rectal artesunate (an artemisinin monotherapy) as a pre-referral treatment; for the purposes of this analysis, patients who received this treatment were dropped (*n* = 22 for Uga2, and *n* = 45 for Uga3). Detailed descriptions of the specific interventions are included in individual study reports, and an overview is presented in Burchett et al. [19].

### Statistical analysis

The following variables were assessed as potential explanatory factors of outcomes of interest: sex, age, mRDT proportion positive as a proxy for endemicity, health care sector, and intervention arm, where individual studies collected data on these variables. Formal meta-analysis was deemed inappropriate due to heterogeneity in study and intervention design. Univariable and multivariable analyses were conducted for each study separately, using logistic regression with robust standard errors to account for clustering by the primary unit of sampling or randomisation. Variables significantly associated with the outcome of interest in univariable analyses (*p* value for Wald’s test < 0.05) were included in multivariable analyses, along with age and sex identified a priori. Statistical analyses were conducted in STATA 14.0 (STATA Corp LP, College Station, TX).

## Results

### Patient characteristics for mRDT-positive patients

ACT Consortium studies recorded data on a total of 562,762 outpatients presenting for health care in Africa. Of these, 106,039 patients, median age 6 years (IQR 2 to 18 years), tested positive by mRDT. Of the mRDT-positive patients, 7426 (7.0%) were not prescribed an ACT antimalarial. The proportion of mRDT-positive patients not prescribed an ACT ranged across sites from 1.3% in Uga3 to 37.1% in Tanz1 (Additional file 1: Table S1).

### Factors associated with ACT non-prescription for mRDT-positive patients

#### Patient age

Overall, 3473/44,539 (7.8%) of patients younger than 5 years old were not prescribed an ACT antimalarial; this proportion was 3833/60,043 (6.4%) for those aged 5 years and older. The proportion varied by site, ranging from 1.3 (in Uga3) to 41.8% (in Tanz1) of mRDT-positive children < 5 not given an ACT antimalarial (Table 3). In two of eight study sites, the odds of ACT non-prescription were significantly higher for younger children compared with older patients (Ghan1 in those under 5 years, and Uga2 in those under 12 months), with a trend towards this association in three additional studies (Cam1, Tanz1, and Uga1). The only exception was Tanz2 where the odds of ACT non-prescription were higher for older patients (Table 3).

**Table 3:** Association of age with non-prescription of ACT among mRDT-positive patients

Project site	Age (years)	Total number (%) of mRDT-positive patients not prescribed ACT by age*	Unadjusted			Adjusted^†^		
			OR	95% CI^‡^	*p* value^‡^	OR	95% CI^‡^	*p* value^‡^
Cam1	< 5	27/94 (28.7)	1.00	Ref.	0.112	1.00	Ref.	0.089
	≥ 5	35/166 (21.1)	0.66	0.40, 1.10		0.64	0.38, 1.07	
Ghan1	< 5	15/406 (3.7)	1.00	Ref.	< 0.001	1.00	Ref.	< 0.001
	≥ 5	14/899 (1.6)	0.41	0.30, 0.57		0.41	0.30, 0.57	
Nige1	< 5	16/42 (38.1)	1.00	Ref.	0.218	1.00	Ref.	0.618
	≥ 5	141/493 (28.6)	0.65	0.33, 1.29		0.85	0.46, 1.59	
Tanz1	< 5	64/153 (41.8)	1.00	Ref.	0.049	1.00	Ref.	0.077
	≥ 5	24/84 (28.6)	0.56	0.31, 1.00		0.55	0.28, 1.07	
Tanz2	< 5	247/1480 (16.7)	1.00	Ref.	< 0.001	1.00	Ref.	< 0.001
	≥ 5	700/2916 (24.0)	1.58	1.31, 1.90		1.42	1.15, 1.76	
Uga1	< 5	3015/37,287 (8.1)	1.00	Ref.	0.081	1.00	Ref.	0.063
	≥ 5	2862/51,473 (5.6)	0.67	0.43, 1.05		0.66	0.42, 1.02	
Uga3	< 5	30/2239 (1.3)	1.00	Ref.	0.917	1.00	Ref.	0.898
	≥ 5	44/3358 (1.3)	0.98	0.64, 1.50		0.97	0.63, 1.49	
Uga2^§^	< 1	11/242 (4.6)	1.00	Ref.	0.017	1.00	Ref.	0.034
	≥ 1	46/3135 (1.5)	0.31	0.12, 0.81		0.32	0.11, 0.92	

*n is number of patients per study site (and endemicity setting) not prescribed ACT among all mRDT-positive patients with complete data for age, gender, endemicity setting, sector, and intervention arm. Total number of mRDT-positive patients not prescribed ACT: *N* = 7291/104,454

^†^All adjusted models included age and gender as a priori variables and where sufficient data available (≥ 10 outcomes per cell), plus all other variables found significant by univariate analyses (*p* < 0.05). Statistical models for each site vary in composition due to differences in study designs. Final regression models for each site include the following variables: Cam1—gender and age; Ghan1—age only; Nige1—gender, age, sector, and endemicity setting; Tanz1—gender, age, and endemicity setting; Tanz2—gender, age, and endemicity setting; Uga1—gender and age; Uga2—gender, age, and endemicity setting; and Uga3—gender and age

^‡^Confidence intervals and *p* value calculated using Wald’s test

^§^Uga2 age categories </≥ 1 due to primary study limited to patients aged under 6 years

#### Malaria endemicity (using mRDT positivity as a proxy)

Prescription practices were compared within primary studies that included sites with varied levels of endemicity (Nige1, Tanz1, Tanz2, Uga2). In these studies, the odds of not being prescribed an ACT were 2 to 32 times higher for patients seeking care in lower-transmission settings as compared to those seeking care in areas of higher transmission (Table 4). Analysis of patient age and endemicity suggested that ACT non-prescription is particularly common for children under age five in lower-transmission settings (Table 4); for example, in Tanz1, 34/110 (31%) of mRDT-positive under-fives in higher transmission settings were not prescribed ACTs, whilst in low-transmission settings, this proportion was 70%.

**Table 4:** Association of endemicity and age with non-prescription of ACT among mRDT-positive patients

Project site	mRDT positivity	Age (years)	Number (%) of mRDT-positive patients not prescribed ACT by proxy endemicity setting*	Unadjusted			Adjusted^†^		
				OR	95% CI^‡^	*p* value^‡^	OR	95% CI^‡^	*p* value^‡^
(i) Effect of endemicity setting on ACT non-prescription in baseline age group (< 5 years)									
Nige1	High	< 5	95/399 (23.8)	1.00	Ref.	0.004	1.00	Ref.	0.077
	Mod–high		62/134 (46.3)	2.76	1.39, 5.45		1.98	0.93, 4.22	
Tanz1	Mod–high	< 5	47/173 (27.2)	1.00	Ref.	0.026	1.00	Ref.	0.027
	Low		41/64 (64.1)	4.78	1.21, 18.93		4.80	1.19, 19.34	
Tanz2	Mod–low	< 5	770/4102 (18.8)	1.00	Ref.	0.001	1.00	Ref.	< 0.001
	Low		177/294 (60.2)	6.55	2.88, 14.85		6.22	2.70, 14.35	
Uga2^§^	Mod–high	< 1	46/3364 (1.4)	1.00	Ref.	< 0.001	1.00	Ref.	< 0.001
	Low		11/35 (31.4)	33.1	11.88, 92.00		32.49	11.36, 92.92	
(ii) Effect of age on ACT non-prescription in differing areas of endemicity									
Nige1	High	< 5	10/26 (38.5)	1.00	Ref.	0.052	1.00	Ref.	0.051
		≥ 5	85/373 (22.8)	0.47	0.22, 1.01		0.47	0.22, 1.00	
	Mod–high^‖^	< 5	6/16 (37.5)	–	–	–	–	–	–
		≥ 5	56/118 (47.5)						
Tanz1	Mod–high	< 5	34/110 (30.9)	1.00	Ref.	0.226	1.00	Ref.	0.221
		≥ 5	13/63 (20.6)	0.58	0.24, 1.40		0.58	0.24, 1.39	
	Low	< 5	30/43 (69.8)	1.00	Ref.	0.132	1.00	Ref.	0.124
		≥ 5	11/21 (52.4)	0.48	0.18, 1.25		0.47	0.18, 1.23	
Tanz2	Mod–high	< 5	212/1429 (14.8)	1.00	Ref.	< 0.001	1.00	Ref.	< 0.001
		≥ 5	558/2673 (20.9)	1.51	1.23, 1.86		1.51	1.23, 1.86	
	Low	< 5	35/51 (68.6)	1.00	Ref.	0.117	1.00	Ref.	0.109
		≥ 5	142/243 (58.4)	0.64	0.37, 1.12		0.67	0.43, 1.09	
Uga2^§^	Mod–high	< 1	10/238 (4.2)	1.00	Ref.	0.012	1.00	Ref.	0.013
		≥ 1	36/3104 (1.2)	0.27	0.10, 0.75		0.27	0.09, 0.75	
	Low^‖^	< 1	1/4 (25.0)	–	–	–	–	–	–
		≥ 1	10/31 (32.3)						

*n is number of patients per study site not prescribed ACT among all mRDT-positive patients with complete data for age, gender, endemicity setting, sector, and intervention arm. Total number of mRDT-positive patients not prescribed ACT: *N* = 7291/104,454

^†^All adjusted models included age and gender as a priori variables and where sufficient data available (≥ 10 outcomes per cell), plus all other variables found significant by univariate analyses (*p* < 0.05). Statistical models for each site vary in composition due to differences in study designs. Final regression models for each site include the following variables: Nige1—gender, age, sector, and endemicity; Tanz1—gender, age, and endemicity setting; Tanz2—gender, age, and endemicity setting; Uga2—gender, age, and endemicity setting; Nige1 (high)—gender and age; Tanz1 (mod–high)—gender and age; Tanz1 (low)—gender and age; Tanz2 (mod–high)—gender and age; Tanz2 (low)—gender, age, and intervention arm; and Uga2 (mod–high)—gender and age. Nige1 (mod–high) and Uga2 (low) had insufficient outcomes in binary age categories to undergo analysis

^‡^Confidence intervals and *p* value calculated using Wald’s test

^§^Uga2 age categories </≥ 1 due to primary study limited to patients aged under 6 years

^‖^Analysis not undertaken due to insufficient number of outcomes

#### Other factors

In most studies, there was no evidence to suggest an association between patient gender and ACT non-prescription. The exception was Uga1, where female patients had 1.14 times the odds of not being prescribed ACT than male patients (*p* = 0.02). There were no differences observed when comparing ACT non-prescription for mRDT-positive patients attending private versus public health facilities (in the single study where it was possible to assess this), or for those randomised to a no/basic training arm versus an enhanced intervention arm (Additional file 1: Table S2).

### Prescription of other medications to mRDT-positive patients

Of the 7426 mRDT-positive patients not prescribed an ACT antimalarial, 4121 (55.5%) patients (ranging from 17.1 [in Tanz1] to 82.6% [in Tanz2] across study sites) were prescribed other, non-ACT antimalarial medications (e.g. amodiaquine, chloroquine, or sulfadoxine-pyrimethamine, as monotherapy or in combination). In four studies (Cam1, Ghan1, Tanz2, Uga1), more than half of the patients with a positive mRDT who were not prescribed an ACT were prescribed a non-ACT antimalarial (Additional file 1: Table S1).

**Fig. 1:**
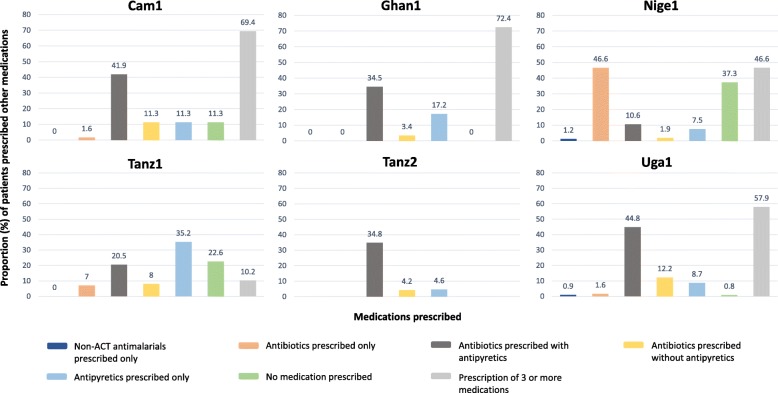
Description of medications prescribed to mRDT-positive patients not prescribed ACT

In mRDT-positive patients who were not prescribed an ACT, at least one antibiotic was prescribed to 3882 (53.2%) of these patients (from 12.4 [Nige1] to 57.0% [Uga1] across study sites; this analysis excludes Uga2 where CHWs only had antimalarials, and Uga3 where other medications were not routinely recorded). A small proportion of patients (*n* = 105, 1.7%) were prescribed only an antibiotic (0 [Ghan1, Tanz2] to 7% [Tanz1]) and no other medication. Antipyretic prescription, with or without other medications, ranged from 52.8 (Nige1) to 93.1% (Ghan1) across studies. For 4.6 (Tanz2) to 35.2% (Tanz1) of patients, antipyretics were the only medication prescribed. mRDT-positive patients prescribed no medication at all ranged from 0 (Ghan1) to 37.3% (Nige1) (Fig. 1). For comparison purposes, the same analysis was conducted for mRDT-positive patients who *were* prescribed ACT (*n* = 98,613). At least one antibiotic was prescribed to 34,573 (38.5%) of these patients (from 16.0 [Ghan1] to 64.5% [Cam1] across study sites). Antipyretic prescription, with or without other medications, ranged from 73.6 (Cam1) to 97.9% (Tanz2) across studies.

### Factors associated with prescription of non-ACT antimalarials and antibiotics

The analysis of factors associated with prescription of non-ACT antimalarials was restricted to six studies due to low outcome numbers in Ghan1 and Tanz1. Only one study, Uga1, showed evidence of an association between patient age and non-ACT antimalarial or antibiotic prescription. In this study, children under age 5 years had higher odds of being prescribed non-ACT antimalarials compared with older patients (adjusted odds ratio [AOR] 0.20, *p* < 0.001) (Additional file 1: Table S3). In contrast, older patients had higher odds of being prescribed an antibiotic (AOR 1.58, *p* < 0.001) (Additional file 1: Table S3). Neither health care sector, nor mRDT positivity as a proxy for malaria endemicity, nor study intervention arm was associated with prescription of non-ACT antimalarials or antibiotics.

## Discussion

Cases of non-severe malaria should have a good outcome if diagnosed and treated in a timely way with the recommended ACT antimalarial; however, malaria is potentially fatal if not treated appropriately. This is particularly true for young children and patients in many low-transmission settings, and in these groups, a proven parasitaemia is very likely to be relevant to the clinical presentation. The current analysis of data from more than 100,000 mRDT-positive outpatients across several sites in 5 African countries found widespread non-treatment with the recommended first-line antimalarial treatment for malaria. This phenomenon was more common in patient groups at highest risk of poor outcomes for malaria infection: younger children (aged < 5 years, or infants < 12 months in one study that enrolled only children under age six) and patients of all ages in lower-transmission settings. In some subgroups, up to 70% of malaria-positive patients were not prescribed an ACT antimalarial; this is potentially very dangerous. Malaria treated early with effective drugs can usually be readily cured; in vulnerable groups, if treated late, or not treated at all, the outcome can be severe or fatal.

At the time of the studies in this analysis, an ACT was recommended as the first-line treatment for malaria test-positive patients in all study areas. Over recent years, substantial efforts have been made to target ACTs to patients who have a parasite-based diagnosis of malaria. The development and deployment of mRDTs in the African region have increased access to confirmatory diagnostics, and in many settings, use of mRDTs has reduced unnecessary antimalarial prescription for mRDT-negative patients. This study suggests, however, that the welcome reduction of over-prescription of antimalarials to patients who test negative for malaria has been accompanied by a reduction in treatment for those who do have malaria, a potentially life-threatening disease. In older patients in high-transmission settings, asymptomatic parasitaemia is common, and finding parasites on testing febrile patients may be coincidental, and not relevant to the aetiology of fever. In low-transmission settings, and in young children prior to the acquisition of appreciable immunity, finding malaria parasites is almost always relevant (the patients have malaria) and outcomes from not treating malaria are potentially serious. It is, therefore, contrary to clinical logic for non-treatment as found here, actually to be higher in these groups.

The primary studies that contributed data to this analysis were conducted between 2007 and 2013, which has two implications. First, in common with most synthesis studies, the data are a few years old and may not reflect current practice. Second, the studies were conducted relatively early in ACT implementation in routine care and at the time of mRDT introduction (by design). It may take time for policy change to translate fully into clinical practice, and whilst there is some evidence that test adherence has improved in some settings with strong supervision or training [30, 31], other evidence suggests that under-treatment of malaria test-positive patients persists (in proportions both similar to, and higher than, those reported in this paper) [32–34]. Based on current data, it would be rash to assume that this under-diagnosis and subsequent under-treatment has simply disappeared since 2013 without further intervention, particularly as the problem of under-treatment in high-risk groups has not been highlighted.

As part of mRDT introduction in the primary studies, training messages advised health care workers to perform a mRDT (or microscopy, where appropriate) and prescribe ACT for positive results. In this analysis, just over half of the test-positive patients who did not receive ACT were prescribed a different, non-recommended antimalarial; even more concerningly, overall, 44.5% were prescribed no antimalarial medication at all. Furthermore, when comparing antibiotic prescription among mRDT-positive patients who were prescribed ACT and those who were not, the proportions of antibiotic prescription were substantially higher among those not prescribed ACT (53.2% vs. 38.5%) [35]. There is no obvious clinical logic to performing a diagnostic test and then ignoring a positive result. mRDTs based on histidine-rich protein 2 (HRP2), which are the predominant mRDT type used in most African contexts including in the study areas analysed here, may detect residual antigenemia persisting after a cleared *P. falciparum* infection, but guidelines typically advise that providers only consider the possibility of a false-positive mRDT result on this basis for a short while after effective treatment of a confirmed infection [2], which presumably would have been a rare case presentation in the study populations. Similarly, asymptomatic parasite carriage does occur, but generally in high-transmission settings and among older children and adults who have developed partial immunity to malaria.

The primary ACT Consortium studies that provided data for this analysis did not record health care workers’ reasons for prescribing certain drugs, and exploring this further would require qualitative studies. Whilst it is therefore not possible to draw direct conclusions from these data about the causes of ACT non-prescription, a number of possible explanatory factors were suggested by Burchett et al. [19], including motivation to perform well in a study context, stability of ACT supplies, and local preferences for different types of antimalarials. More broadly, other previous work has identified some factors that are associated with non-adherence to test results and/or treatment guidelines, including distrust in the test or test result [36–39], patient demands or preferences for a particular medication [19, 40–43], perceptions of low drug efficacy [41], staff workload [44], financial incentives [42], level of health care worker [41, 45, 46], affordability and accessibility of non-recommended therapies [47, 48], and rationing of medications [19, 49]. Stock-outs of weight-specific drug packs can also lead to inappropriate prescription of medications [45, 50], whilst lack of knowledge on how to prescribe second-line drugs can lead to not prescribing them at all [51]. The availability of antimalarials has been shown to influence prescribing patterns [45, 47, 52], and health care workers are often restricted to prescribing what is available to them. With the exception of Tanz1 which evaluated “real-world” mRDT implementation, ACT stocks were generally maintained in the study areas during the primary ACT Consortium studies; it is possible, however, that some combination of these factors, which are more common outside of optimal study conditions, contributed to the prescribing behaviours seen in this analysis. For example, the prescription of older, non-ACT antimalarials may have persisted due to the continued availability of unused stock during the time period of the studies, which was shortly after the introduction of ACTs in many study areas.

Current guidelines to “test before treating” for malaria [2] have been criticised for not accounting for variation in malaria transmission and epidemiology [53]. Partial immunity to malaria is more common among individuals with higher levels of exposure, in whom fever symptoms may not be attributable to incidental parasitemia detected by diagnostic tests [48, 49, 53].

A strength of this analysis is that it draws on data from a large number of routine outpatient visits in eight primary studies in diverse malaria-endemic settings in Africa, allowing analysis of prescribing practices across representative health care contexts. Although less commonly reported, non-adherence in prescribing for mRDT-*positive* patients has been acknowledged previously [18, 19]; this analysis quantitatively explores a gap in knowledge about prescribing practices by identifying specific populations to which the risk of inappropriate treatment is greatest, and demonstrating that the probability of under-treatment is greatest in those in whom the *risk* of under-treatment is also greatest.

This analysis was subject to a number of limitations. Firstly, because at the time of study initiation over-diagnosis and over-treatment were almost universal, non-adherence to positive mRDT results was not anticipated when the ACT Consortium primary studies were designed so the studies did not collect explanatory qualitative information on this phenomenon. It is not possible, therefore, to discern from these data *why* providers did not always prescribe according to positive mRDT results. Similarly, a number of factors described in previous reports (as summarised above) that may influence prescribing practices were not consistently assessed in ACT Consortium studies and were not included in this analysis. Thirdly, whilst the primary studies used similar methodologies, there were substantial differences in study design and data collected, precluding formal meta-analysis. Furthermore, not all studies recorded details of non-ACT medications prescribed, limiting analysis of the secondary outcomes to data from a smaller number of sites. We cannot exclude the possibility that some patients had already started on antimalarials at the time of presentation, or reported having them at home, and so did not receive a prescription. Finally, the proportion of patients testing positive for malaria is only a crude proxy for malaria endemicity, but this is more likely to dilute any effect of endemicity rather than inflate it.

Febrile patients make up a large proportion of all health care seeking in Africa, and until recently, empiric, syndromic treatment was the norm, with inevitable misdiagnosis and, in particular, over-diagnosis of malaria. Encouraging health workers to perform a malaria test, and to adhere to negative test results, has been a remarkable public health advance in malaria and fever case management. However, these advances may come at a serious clinical cost if true malaria cases in vulnerable groups at risk of poor outcomes are left untreated. It is well known that changing one health care practice can have unintended consequences for other practices; for example, mRDT introduction also tends to drive more prescription of antibiotics [35, 54], and it can also influence patient satisfaction with care and affect the likelihood of treatment seeking [54, 55]. The introduction of diagnostic technology is not always straightforward, and future interventions should emphasise the importance of following recommendations for both test-positive and test-negative patients. Case management guidelines need to be accessible, unambiguous, and consistently used, and must reinforce the need to treat malaria test-positive patients with an ACT, especially in vulnerable groups [56].

## Conclusion

This analysis shows that in several settings, significant numbers of malaria test-positive patients were not prescribed antimalarials and in particular that this was true for the vulnerable groups of young children and those in low-transmission settings. Those responsible for clinical services in malaria-endemic areas need to ensure that all mRDT-positive patients are prescribed, and receive, locally recommended treatment, and that providers have reliable access to effective antimalarials and the confidence to trust mRDT results and prescribe accordingly.

## Supplementary information

**Additional file 1: Table S1.** Number (%) of mRDT-positive patients i) not prescribed ACT and ii) prescribed other medications. **Table S2.** Risk factors associated with non-prescription of ACT in mRDT-positive patients not prescribed ACT. **Table S3.** Risk factors associated with prescription of medications in mRDT-positive patients not prescribed ACT.

## Data Availability

All individual study data used in the analysis of this study are available at the ACT Consortium data repository (https://actc.lshtm.ac.uk/) [57] or from the authors on request. All data generated or analysed during this study are included in this article and its supplementary information files.
