# Supplementary material for: Patients with positive malaria tests not given artemisinin-based combination therapies: a research synthesis describing under-prescription of antimalarial medicines in Africa
Source: BMC Med. 2020 Jan 30;18:17. doi: 10.1186/s12916-019-1483-6 (PMC6990477; doi:10.1186/s12916-019-1483-6)
Supplement: Supplementary file 1 — Additional file 1: Table S1. Number (%) of mRDT-positive patients i) not prescribed ACT and ii) prescribed other medications. Table S2. Risk factors associated with non-prescription of ACT in mRDT-positive patients not prescribed ACT. Table S3. Risk factors associated with prescription of medications in mRDT-positive patients not prescribed ACT. [file 12916_2019_1483_MOESM1_ESM.docx]

**Table S1 | Number (%) of mRDT-positive patients i) not prescribed ACT and ii) prescribed other medications**

| **Project site** | **Indicators of interest** | **Number (%) of mRDT-positive patients not prescribed ACT by variable subset** | **Number (%) and type of other medication prescribed to mRDT-positive patients not prescribed ACT**‡ | | |
| --- | --- | --- | --- | --- | --- |
|  |  |  | **non-ACT Antimalarials** | **Antibiotics** | **Antipyretics** |
| **Cam1** | **Total** | **62/260 (23.9)** | **38/62 (61.3)** | **33/62 (53.2)** | **45/62 (72.6)** |
| Gender | Male  Female | 27/123 (22.0)  35/137 (25.6) | 12/27 (44.4)  26/35 (68.4) | 13/27 (48.2)  20/35 (57.1) | 18/27 (66.7)  27/35 (77.1) |
| Age (years) | <5  ≥5 | 27/94 (28.7)  35/166 (21.1) | 15/27 (55.6)  23/35 (65.7) | 14/27 (51.9)  19/35 (54.3) | 17/27 (63.0)  28/35 (80.0) |
| Intervention arm | No/basic training  BC arm | 24/98 (24.5)  38/162 (23.5) | 13/24 (54.2)  25/38 (65.8) | 8/24 (33.3)  25/38 (65.8) | 16/24 (66.7)  29/38 (76.3) |
| **Ghan1** | **Total** | **29/1,305 (2.2)** | **17/29 (58.6)** | **11/29 (37.9)** | **27/29 (93.1)** |
| Gender | Male  Female | 9/596 (1.5)  20/709 (2.8) | 3/9 (33.3)  14/20 (70.0) | 6/9 (66.7)  5/20 (25.0) | 8/9 (88.9)  19/20 (95.0) |
| Age (years) | <5  ≥5 | 15/406 (3.7)  14/899 (1.6) | 10/15 (66.7)  7/14 (50.0) | 4/15 (26.7)  7/14 (50.0) | 13/15 (86.7)  14/14 (100.0) |
| **Nige1** | **Total** | **161/544 (29.6)** | **77/161 (47.8)** | **20/161 (12.4)** | **85/161 (52.8)** |
| Gender* | Male  Female | 76/252 (30.2)  84/286 (29.4) | 41/76 (54.0)  36/84 (42.9) | 11/76 (14.5)  9/84 (10.7) | 46/76 (60.5)  39/84 (46.3) |
| Age (years)* | <5  ≥5 | 16/42 (38.1)  141/493 (28.6) | 5/16 (31.3)  71/141 (50.4) | 2/16 (12.5)  18/141 (12.8) | 9/16 (56.3)  74/141 (52.5) |
| Sector | Public  Private | 74/172 (43.0)  87/372 (23.4) | 35/74 (47.3)  42/87 (48.3) | 16/74 (21.6)  4/87 (4.6) | 51/74 (68.9)  34/87 (39.1) |
| Endemicity (by proxy) | High  Mod-High | 99/407 (24.3)  62/134 (46.3) | 37/99 (37.4)  40/62 (64.5) | 7/99 (7.1)  13/62 (21.0) | 37/99 (37.4)  48/62 (77.4) |
| Intervention arm | No/basic training  BC arm  BC + CS arm | 77/309 (24.9)  46/140 (32.9)  38/95 (40.0) | 29/77 (37.7)  25/46 (54.4)  23/38 (60.5) | 3/77 (3.9)  11/46 (23.9)  6/38 (15.8) | 30/77 (39.0)  32/46 (69.6)  23/38 (60.5) |
| **Tanz1** | **Total** | **88/237 (37.1)** | **15/88 (17.1)** | **25/87* (28.4)** | **58/87* (66.7)** |
| Gender | Male  Female | 37/105 (35.2)  51/132 (38.6) | 3/37 (8.1)  12/51 (23.5) | 9/37 (24.3)  16/50* (32.0) | 26/37 (70.3)  32/50* (64.0) |
| Age (years) | <5  ≥5 | 64/153 (41.8)  24/84 (28.6) | 12/64 (18.8)  3/24 (12.5) | 21/64 (32.8)  4/23 (17.4) | 43/64 (67.2)  15/23 (65.2) |
| Endemicity (by proxy) | Mod-High  Low | 47/173 (27.2)  41/64 (64.1) | 9/47 (19.2)  6/41 (14.6) | 12/47 (25.5)  13/40 (32.5) | 28/47 (59.6)  30/40 (75.0) |
| **Tanz2** | **Total** | **949/4,400 (21.6)** | **784/949 (82.6)** | **370/949 (39.0)** | **889/949 (93.7)** |
| Gender | Male  Female | 424/2,037 (20.8)  525/2,363 (22.2) | 361/424 (85.1)  423/525 (80.6) | 164/424 (38.7)  206/525 (39.2) | 404/424 (95.3)  485/525 (92.4) |
| Age (years)* | <5  ≥5 | 247/1,480 (16.7)  700/2,916 (24.0) | 198/247 (80.2)  584/700 (83.4) | 102/247 (41.3)  267/700 (38.1) | 225/247 (91.1)  662/700 (94.6) |
| Endemicity (by proxy) | Mod-Low  Low | 771/4,105 (18.8)  178/295 (60.3) | 623/771 (80.8)  161/178 (90.5) | 293/771 (38.0)  77/178 (43.3) | 729/771 (94.6)  160/178 (89.9) |
| Intervention arm | No/basic training  BC arm  BC + CS arm | 309/1,455 (21.2)  335/1,696 (19.8)  305/1,249 (24.4) | 246/309 (79.6)  281/335 (83.9)  257/305 (84.3) | 112/309 (36.3)  133/335 (39.7)  125/305 (41.0) | 294/309 (95.2)  321/335 (95.8)  274/305 (89.8) |
| **Uga1** | **Total** | **6,006/90,269 (6.7)** | **3,190/6,006 (53.1)** | **3,423/6,006 (57.0)** | **4,851/6,006 (80.8)** |
| Gender* | Male  Female | 2,302/36,167 (6.4)  3,688/53,958 (6.8) | 1,396/2,302 (60.6)  1,784/3,688 (48.4) | 1,253/2,302 (54.4)  2,160/3,688 (58.6) | 1,805/2,302 (78.4)  3,035/3,688 (82.3) |
| Age (years)* | <5  ≥5 | 3,024/37,339 (8.1)  2,868/51,536 (5.6) | 2,197/3,024 (72.7)  962/2,868 (33.5) | 1,549/3,024 (51.2)  1,798/2,868 (62.7) | 2,397/3,024 (79.3)  2,355/2,868 (82.1) |
| Intervention arm | No/basic training  BC arm | 455/8,910 (5.1)  5,551/81,359 (6.8) | 263/455 (57.8)  2,927/5,551 (52.7) | 241/455 (53.0)  3,182/5,551 (57.3) | 384/455 (84.4)  4,467/5,551 (80.5) |
| **Uga2** | **Total** | **57/3,399 (1.7)** | **-*** | **-*** | **-*** |
| Gender* | Male  Female | 30/1,792 (1.7)  27/1,592 (1.7) | -  - | -  - | -  - |
| Age (years)* | <1  ≥1 | 11/242 (4.6)  46/3,135 (1.5) | -  - | -  - | -  - |
| Endemicity (by proxy) | Mod-High  Low | 46/3,364 (1.4)  11/35 (31.4) | -  - | -  - | -  - |
| **Uga3** | **Total** | **74/5,625 (1.3)** | **-*** | **-*** | **-*** |
| Gender* | Male  Female | 31/2,703 (1.2)  43/2,915 (1.5) | -  - | -  - | -  - |
| Age (years)* | <5  ≥5 | 30/2,239 (1.3)  44/3,358 (1.3) | -  - | -  - | -  - |
| **Total** |  | **7,426/106,039 (7.0)** | **4,121/7,295* (56.5)** | **3,882/7,294* (53.2)** | **5,955/7,294* (81.6)** |
| BC = enhanced training arm with behaviour change component; BC + CS = enhanced training arm with behaviour change and community sensitisation components | | | | | |
| - Data were not collected on antibiotics (Uga2), antipyretics (Uga2) | | | | | |
| * Denominator differs from reported total (mRDT-positive patients not prescribed ACT) due to: 1) missing data: in all mRDT+ patients data missing for gender (172), age (1,457); in mRDT+ patients not prescribed ACT data missing for gender (17), age (120), antibiotic prescription (1), and antipyretic prescription (1); or, 2) where primary studies did not prescribe or routinely record prescription of other medications and were thereby excluded from this analysis (Uga2, N=57 and Uga3, N=74) | | | | | |
| † N=106,039 (total number of patients testing positive by mRDT, and excluding those prescribed malaria suppositories: Uga2, n=22 ; Uga3, n=45) | | | | | |
| ‡ N=7,426 (total number of patients testing positive by mRDT who were not prescribed ACT) | | | | | |
| § Uga2 age categories </≥ 1 due to primary study limited to patients aged under 6 years | | | | | |

**Table S2. Risk factors associated with non-prescription of ACT in mRDT-positive patients not prescribed ACT**

| Project site  Indicators of interest | | Unadjusted | | | Adjusted† | | |
| --- | --- | --- | --- | --- | --- | --- | --- |
|  |  | **OR** | **95% CI‡** | **p-value‡** | **OR** | **95% CI‡** | **p-value‡** |
| Cam1 | **n (62)*** | | | | | | |
| Gender | Male | 1.00 | (Ref.) | 0.501 | 1.00 | (Ref.) | 0.409 |
|  | Female | 1.22 | (0.68, 2.18) |  | 1.29 | (0.71, 2.35) |  |
| Age (Years) | <5 | 1.00 | (Ref.) | 0.112 | 1.00 | (Ref.) | 0.089 |
|  | ≥5 | 0.66 | (0.40, 1.10) |  | 0.64 | (0.38, 1.07) |  |
| Intervention | No/Basic training | 1.00 | (Ref.) | 0.905 | --- | --- | --- |
|  | BC arm | 0.94 | (0.37, 2.40) |  |  |  |  |
| Ghan1 | **n (29)** | | | | | | |
| Age (Years) | <5 | 1.00 | (Ref.) | <0.001 | 1.00 | (Ref.) | <0.001 |
|  | ≥5 | 0.41 | (0.30, 0.57) |  | 0.41 | (0.30, 0.57) |  |
| Nige1 | **n (157)** | | | | | | |
| Gender | Male | 1.00 | (Ref.) | 0.614 | 1.00 | (Ref.) | 0.302 |
|  | Female | 0.93 | (0.69, 1.24) |  | 0.85 | (0.62, 1.16) |  |
| Age (Years) | <5 | 1.00 | (Ref.) | 0.218 | 1.00 | (Ref.) | 0.618 |
|  | ≥5 | 0.65 | (0.33, 1.29) |  | 0.85 | (0.46, 1.59) |  |
| Sector | Public | 1.00 | (Ref.) | 0.011 | 1.00 | (Ref.) | 0.280 |
|  | Private | 0.40 | (0.20, 0.81) |  | 0.62 | (0.25, 1.49) |  |
| Endemicity | High | 1.00 | (Ref.) | 0.004 | 1.00 | (Ref.) | 0.077 |
| (by proxy) | Mod-high | 2.76 | (1.39, 5.45) |  | 1.98 | (0.93, 4.22) |  |
| Intervention | No/Basic training | 1.00 | (Ref.) | 0.135 | --- | --- | --- |
|  | BC arm | 1.55 | (0.68, 3.53) |  |  |  |  |
|  | BC+CS arm | 2.06 | (0.95, 4.47) |  |  |  |  |
| Tanz1 | **n (88)** | | | | | | |
| Gender | Male | 1.00 | (Ref.) | 0.556 | 1.00 | (Ref.) | 0.807 |
|  | Female | 1.16 | (0.71, 1.88) |  | 1.07 | (0.63, 1.80) |  |
| Age (Years) | <5 | 1.00 | (Ref.) | 0.049 | 1.00 | (Ref.) | 0.077 |
|  | ≥5 | 0.56 | (0.31, 1.00) |  | 0.55 | (0.28, 1.07) |  |
| Endemicity | Mod-high | 1.00 | (Ref.) | 0.026 | 1.00 | (Ref.) | 0.027 |
| (by proxy) | Low | 4.78 | (1.21, 18.93) |  | 4.80 | (1.19, 19.34) |  |
| Tanz2 | **n (947)** | | | | | | |
| Gender | Male | 1.00 | (Ref.) | 0.278 | 1.00 | (Ref.) | 0.516 |
|  | Female | 1.09 | (0.94, 1.25) |  | 1.06 | (0.90, 1.25) |  |
| Age (Years) | <5 | 1.00 | (Ref.) | <0.001 | 1.00 | (Ref.) | <0.001 |
|  | ≥5 | 1.58 | (1.31, 1.90) |  | 1.42 | (1.15, 1.76) |  |
| Endemicity | Mod-low | 1.00 | (Ref.) | 0.001 | 1.00 | (Ref.) | <0.001 |
| (by proxy) | Low | 6.55 | (2.88, 14.85) |  | 6.22 | (2.70, 14.35) |  |
| Intervention | No/Basic training | 1.00 | (Ref.) | 0.903 | --- | --- | --- |
|  | BC arm | 0.91 | (0.37, 2.25) |  |  |  |  |
|  | BC+CS arm | 1.19 | (0.38, 3.72) |  |  |  |  |
| Uga1 | **n (5,877)** | | | | | | |
| Gender | Male | 1.00 | (Ref.) | 0.350 | 1.00 | (Ref.) | 0.020 |
|  | Female | 1.08 | (0.92, 1.27) |  | 1.14 | (1.02, 1.28) |  |
| Age (Years) | <5 | 1.00 | (Ref.) | 0.081 | 1.00 | (Ref.) | 0.063 |
|  | ≥5 | 0.67 | (0.43, 1.05) |  | 0.66 | (0.42, 1.02) |  |
| Intervention | No/Basic training | 1.00 | (Ref.) | 0.347 | --- | --- |  |
|  | BC arm | 1.35 | (0.73, 2.50) |  |  |  |  |
| Uga2 | **n (57)** | | | | | | |
| Gender | Male | 1.00 | (Ref.) | 0.961 | 1.00 | (Ref.) | 0.985 |
|  | Female | 1.01 | (0.60, 1.72) |  | 1.00 | (0.58, 1.74) |  |
| Age (Years) | <1 | 1.00 | (Ref.) | 0.017 | 1.00 | (Ref.) | 0.034 |
|  | ≥1 | 0.31 | (0.12, 0.81) |  | 0.32 | (0.11, 0.92) |  |
| Endemicity | Mod-high | 1.00 | (Ref.) | <0.001 | 1.00 | (Ref.) | <0.001 |
| (by proxy) | Low | 33.1 | (11.88, 92.00) |  | 32.49 | (11.36, 92.92) |  |
| Uga3 | **n (74)** | | | | | | |
| Gender | Male | 1.00 | (Ref.) | 0.203 | 1.00 | (Ref.) | 0.210 |
|  | Female | 1.29 | (0.87, 1.91) |  | 1.29 | (0.87, 1.91) |  |
| Age (Years) | <5 | 1.00 | (Ref.) | 0.917 | 1.00 | (Ref.) | 0.898 |
|  | ≥5 | 0.98 | (0.64, 1.50) |  | 0.97 | (0.63, 1.49) |  |
| BC = enhanced training arm with behaviour change components; CS = enhanced training arm with community sensitisation components | | | | | | | |
| * n is number of patients per study site not prescribed ACT among all mRDT-positive patients with complete data for age, Gender, endemicity setting, sector, and intervention arm. Total number of mRDT-positive patients not prescribed ACT: N=7,291 | | | | | | | |
| † All adjusted models included age and Gender as a priori variables and where sufficient data available (≥10 outcomes per cell), plus all other variables found significant by univariate analyses (p<0.05). Statistical models for each site vary in composition due to differences in study designs. Final regression models including variables listed in adjusted column for each study site, respectively | | | | | | | |
| ‡ Confidence intervals and p-value calculated using Wald’s test | | | | | | | |

**Table S3 | Risk factors associated with prescription of medications in mRDT-positive patients not prescribed ACT**

| Project site  Indicators of interest | | Unadjusted | | | Adjusted† | | |
| --- | --- | --- | --- | --- | --- | --- | --- |
|  |  | **OR** | **95% CI‡** | **p-value‡** | **OR** | **95% CI‡** | **p-value‡** |
| i) Prescription of non-ACT antimalarials | | | | | | | |
| Cam1 | **n (38)** | | | | | | |
| Age (Years) | <5 | 1.00 | (Ref.) | 0.403 | 1.00 | (Ref.) | 0.403 |
|  | ≥5 | 1.53 | (0.56, 4.17) |  | 1.53 | (0.56, 4.17) |  |
| Intervention | No/Basic training | 1.00 | (Ref.) | 0.354 | --- | --- | --- |
|  | BC arm | 1.63 | (0.58, 4.56) |  |  |  |  |
| Nige1 | **n (76)** | | | | | | |
| Gender | Male | 1.00 | (Ref.) | 0.172 | 1.00 | (Ref.) | 0.172 |
|  | Female | 0.65 | (0.35, 1.21) |  | 0.65 | (0.35, 1.21) |  |
| Sector | Public | 1.00 | (Ref.) | 0.987 | --- | --- | --- |
|  | Private | 0.99 | (0.17, 5.83) |  |  |  |  |
| Endemicity | High | 1.00 | (Ref.) | 0.145 | --- | --- | --- |
| (as proxy) | Mod-high | 2.98 | (0.69, 12.95) |  |  |  |  |
| Intervention | No/Basic training | 1.00 | (Ref.) | 0.620 | --- | --- | --- |
|  | BC arm | 2.05 | (0.38, 11.15) |  |  |  |  |
|  | BC + CS arm | 2.52 | (0.37, 17.31) |  |  |  |  |
| Tanz2 | **n (782)** | | | | | | |
| Gender | Male | 1.00 | (Ref.) | 0.076 | 1.00 | (Ref.) | 0.054 |
|  | Female | 0.72 | (0.50, 1.04) |  | 0.70 | (0.49, 1.01) |  |
| Age (Years) | <5 | 1.00 | (Ref.) | 0.391 | 1.00 | (Ref.) | 0.303 |
|  | ≥5 | 1.25 | (0.75, 2.06) |  | 1.29 | (0.79, 2.11) |  |
| Endemicity | Mod-low | 1.00 | (Ref.) | 0.231 | --- | --- | --- |
| (as proxy) | Low | 2.24 | (0.60, 8.38) |  |  |  |  |
| Intervention | No/Basic training | 1.00 | (Ref.) | 0.808 | --- | --- | --- |
|  | BC arm | 1.33 | (0.50, 3.53) |  |  |  |  |
|  | BC + CS arm | 1.37 | (0.40, 4.64) |  |  |  |  |
| Uga1 | **n (3,150)** | | | | | | |
| Gender | Male | 1.00 | (Ref.) | <0.001 | 1.00 | (Ref.) | 0.102 |
|  | Female | 0.61 | (0.50, 0.73) |  | 0.84 | (0.69, 1.03) |  |
| Age (Years) | <5 | 1.00 | (Ref.) | <0.001 | 1.00 | (Ref.) | <0.001 |
|  | ≥5 | 0.19 | (0.14, 0.26) |  | 0.20 | (0.14, 0.27) |  |
| Intervention | No/Basic training | 1.00 | (Ref.) | 0.750 | --- | --- | --- |
|  | BC arm | 0.83 | (0.27, 2.59) |  |  |  |  |
| ii) Prescription of antibiotics | | | | | | | |
| Cam1 | **n (33)** | | | | | | |
| Gender | Male | 1.00 | (Ref.) | 0.470 | 1.00 | (Ref.) | 0.475 |
|  | Female | 1.44 | (0.54, 3.83) |  | 1.45 | (0.52, 4.03) |  |
| Age (Years) | <5 | 1.00 | (Ref.) | 0.825 | 1.00 | (Ref.) | 0.948 |
|  | ≥5 | 1.10 | (0.46, 2.62) |  | 0.97 | (0.39, 2.42) |  |
| Tanz1 | **n (25)** | | | | | | |
| Endemicity | Mod-high | 1.00 | (Ref.) | 0.499 | --- | --- | --- |
| (as proxy) | Low | 1.40 | (0.52, 3.76) |  |  |  |  |
| Tanz2 | **n (369)** | | | | | | |
| Gender | Male | 1.00 | (Ref.) | 0.867 | 1.00 | (Ref.) | 0.801 |
|  | Female | 1.02 | (0.79, 1.32) |  | 1.03 | (0.80, 1.34) |  |
| Age (Years) | <5 | 1.00 | (Ref.) | 0.458 | 1.00 | (Ref.) | 0.447 |
|  | ≥5 | 0.88 | (0.62, 1.24) |  | 0.87 | (0.62, 1.24) |  |
| Endemicity | Mod-low | 1.00 | (Ref.) | 0.288 | --- | --- | --- |
| (as proxy) | Low | 1.26 | (0.82, 1.93) |  |  |  |  |
| Intervention | No/Basic training | 1.00 | (Ref.) | 0.869 | --- | --- | --- |
|  | BC arm | 1.15 | (0.44, 3.02) |  |  |  |  |
|  | BC + CS arm | 1.23 | (0.56, 2.68) |  |  |  |  |
| Uga1 | **n (3,338)** | | | | | | |
| Gender | Male | 1.00 | (Ref.) | <0.001 | 1.00 | (Ref.) | 0.081 |
|  | Female | 1.18 | (1.09, 1.28) |  | 1.06 | (0.99, 1.14) |  |
| Age (Years) | <5 | 1.00 | (Ref.) | <0.001 | 1.00 | (Ref.) | <0.001 |
|  | ≥5 | 1.60 | (1.39, 1.84) |  | 1.58 | (1.37, 1.81) |  |
| Intervention | No/Basic training | 1.00 | (Ref.) | 0.617 | --- | --- | --- |
|  | BC arm | 1.16 | (0.64, 2.10) |  |  |  |  |
| iii) Prescription of antipyretics | | | | | | | |
| Nige1 | **n (83)** | | | | | | |
| Gender | Male | 1.00 | (Ref.) | 0.028 | 1.00 | (Ref.) | 0.015 |
|  | Female | 0.55 | (0.32, 0.94) |  | 0.54 | (0.33, 0.89) |  |
| Sector | Public | 1.00 | (Ref.) | 0.108 | --- | --- | --- |
|  | Private | 0.28 | (0.06, 1.32) |  |  |  |  |
| Endemicity | High | 1.00 | (Ref.) | 0.015 | 1.00 | (Ref.) | 0.019 |
| (as proxy) | Mod-high | 5.88 | (1.41, 24.58) |  | 5.91 | (1.33, 26.21) |  |
| Intervention | No/Basic training | 1.00 | (Ref.) | 0.268 | --- | --- | --- |
|  | BC arm | 4.04 | (0.67, 24.27) |  |  |  |  |
|  | BC + CS arm | 2.52 | (0.42, 15.15) |  |  |  |  |
| Tanz1 | **n (58)** | | | | | | |
| Gender | Male | 1.00 | (Ref.) | 0.558 | 1.00 | (Ref.) | 0.558 |
|  | Female | 0.75 | (0.29, 1.95) |  | 0.75 | (0.29, 1.95) |  |
| Endemicity | Mod-high | 1.00 | (Ref.) | 0.445 | --- | --- | --- |
| (as proxy) | Low | 2.04 | (0.33, 12.62) |  |  |  |  |
| Tanz2 | **n (887)** | | | | | | |
| Gender | Male | 1.00 | (Ref.) | 0.054 | 1.00 | (Ref.) | 0.027 |
|  | Female | 0.60 | (0.35, 1.01) |  | 0.56 | (0.34, 0.94) |  |
| Age (Years) | <5 | 1.00 | (Ref.) | 0.024 | 1.00 | (Ref.) | 0.006 |
|  | ≥5 | 1.70 | (1.07, 2.70) |  | 1.91 | (1.21, 3.03) |  |
| Endemicity | Mod-low | 1.00 | (Ref.) | 0.005 | 1.00 | (Ref.) | 0.292 |
| (as proxy) | Low | 0.51 | (0.32, 0.81) |  | 0.81 | (0.55, 1.20) |  |
| Intervention | No/Basic training | 1.00 | (Ref.) | 0.010 | 1.00 | (Ref.) | 0.041 |
|  | BC arm | 1.17 | (0.39, 3.52) |  | 1.22 | (0.41, 3.70) |  |
|  | BC + CS arm | 0.45 | (0.23, 0.87) |  | 0.50 | (0.25, 1.01) |  |
| Uga1 | **n (4,742)** | | | | | | |
| Gender | Male | 1.00 | (Ref.) | 0.005 | 1.00 | (Ref.) | 0.007 |
|  | Female | 1.28 | (1.08, 1.53) |  | 1.24 | (1.06, 1.46) |  |
| Age (Years) | <5 | 1.00 | (Ref.) | 0.278 | 1.00 | (Ref.) | 0.423 |
|  | ≥5 | 1.20 | (0.86, 1.67) |  | 1.14 | (0.82, 1.59) |  |
| Intervention | No/Basic training | 1.00 | (Ref.) | 0.149 | --- | --- | --- |
|  | BC arm | 0.75 | (0.50, 1.11) |  |  |  |  |
| iv) Prescription of antihelminths | | | | | | | |
| Uga1 | **n (721)** | | | | | | |
| Gender | Male | 1.00 | (Ref.) | <0.001 | 1.00 | (Ref.) | 0.607 |
|  | Female | 1.32 | (1.17, 1.48) |  | 1.04 | (0.90, 1.20) |  |
| Age (Years) | <5 | 1.00 | (Ref.) | <0.001 | 1.00 | (Ref.) | <0.001 |
|  | ≥5 | 2.97 | (1.94, 4.55) |  | 2.95 | (1.90, 4.57) |  |
| Intervention | No/Basic training | 1.00 | (Ref.) | 0.064 | --- | --- | --- |
|  | BC arm | 2.29 | (0.95, 5.49) |  |  |  |  |
| BC = enhanced training arm with behaviour change components; CS = enhanced training arm with community sensitisation components | | | | | | | |
| * n is number of patients per study site prescribed other medications among all mRDT-positive patients not prescribed ACT with complete data for age, gender, endemicity setting, sector, and intervention arm. Uga2 and Uga3 did not prescribe or routinely record prescription of other medications and were thereby excluded from this analysis. Total number (N) of mRDT-positive patients/not prescribed ACT but prescribed other medications: non-ACT antimalarials: N=4,078; antibiotics: N=3,796; antipyretics: N=5,842; antihelminths: N=731; antifungals: N=5) | | | | | | | |
| † All adjusted models included age and gender as a priori variables and where sufficient data available (≥10 outcomes per cell), plus all other variables found significant by univariate analyses (p<0.05). Statistical models for each site vary in composition due to differences in study designs. Final regression models including variables listed in adjusted column for each study site, respectively | | | | | | | |
| ‡ Confidence intervals and p-value calculated using Wald’s test | | | | | | | |
